# Automatic segmentation model of intercondylar fossa based on deep learning: a novel and effective assessment method for the notch volume

**DOI:** 10.1186/s12891-022-05378-7

**Published:** 2022-05-06

**Authors:** Mifang Li, Hanhua Bai, Feiyuan Zhang, Yujia Zhou, Qiuyu Lin, Quan Zhou, Qianjin Feng, Lingyan Zhang

**Affiliations:** 1 Southern Medical University, 1838 shatai Road, Baiyun District, Guangzhou, 510515 Guangdong province China; 2 Department of Medical Imaging, Longgang Central Hospital of Shenzhen, 6082 Longgang Avenue, Longgang District, Shenzhen, 518116 Guangdong province China; 3 Department of Medical Imaging, The Third Affiliated Hospital, Southern Medical University, 183 Zhongshan Avenue West, Tianhe District, Guangzhou, 510630 Guangdong province China; 4 Department of Biomedical Engineering, Southern Medical University, 1838 shatai Road, Baiyun District, Guangzhou, 510515 Guangdong province China; 5 Guangdong Provincial Key Laboratory of Medical Image Processing, Southern Medical University, 1838 shatai Road, Baiyun District, Guangzhou, 510515 Guangdong province China; 6 Guangdong Province Engineering Laboratory for Medical Imaging and Diagnostic Technology, Southern Medical University, 1838 shatai Road, Baiyun District, Guangzhou, 510515 Guangdong province China

**Keywords:** Anterior cruciate ligament injury, Deep learning, Magnetic resonance imaging, Intercondylar fossa

## Abstract

**Background:**

Notch volume is associated with anterior cruciate ligament (ACL) injury. Manual tracking of intercondylar notch on MR images is time-consuming and laborious. Deep learning has become a powerful tool for processing medical images. This study aims to develop an MRI segmentation model of intercondylar fossa based on deep learning to automatically measure notch volume, and explore its correlation with ACL injury.

**Methods:**

The MRI data of 363 subjects (311 males and 52 females) with ACL injuries incurred during non-contact sports and 232 subjects (147 males and 85 females) with intact ACL were retrospectively analyzed. Each layer of intercondylar fossa was manually traced by radiologists on axial MR images. Notch volume was then calculated. We constructed an automatic segmentation system based on the architecture of Res-UNet for intercondylar fossa and used dice similarity coefficient (DSC) to compare the performance of segmentation systems by different networks. Unpaired t-test was performed to determine differences in notch volume between ACL-injured and intact groups, and between males and females.

**Results:**

The DSCs of intercondylar fossa based on different networks were all more than 0.90, and Res-UNet showed the best performance. The notch volume was significantly lower in the ACL-injured group than in the control group (6.12 ± 1.34 cm^3^ vs. 6.95 ± 1.75 cm^3^, *P* < 0.001). Females had lower notch volume than males (5.41 ± 1.30 cm^3^ vs. 6.76 ± 1.51 cm^3^, *P* < 0.001). Males and females who had ACL injuries had smaller notch than those with intact ACL (*p* < 0.001 and *p* < 0.005). Men had larger notches than women, regardless of the ACL injuries (*p* < 0.001).

**Conclusion:**

Using a deep neural network to segment intercondylar fossa automatically provides a technical support for the clinical prediction and prevention of ACL injury and re-injury after surgery.

## Background

Anterior cruciate ligament (ACL) injury is related to many risk factors, which include environment, anatomical structure, hormone, and biomechanics [1–3]. Being able to anticipate these risks and take corresponding measures, can reduce the risk of ACL injury and re-injury after surgery [4–6]. Therefore, these risk factors should be studied [1, 2].

Subjects with lower femoral intercondylar fossa volume had significantly increased risk of non-contact ACL injury regardless of gender or shape of intercondylar fossa [7–10]. Fung et al. [11] used a 3D mathematical model and confirmed that ACL is in close contact with the femoral intercondylar fossa during knee movement. When subjected to anterior shear force, the anterior internal bundle of ACL may hit the medial side of the lateral condyle of femur, resulting in ACL injury. The narrow intercondylar fossa is likely to produce additional mechanical stress on the ligament, thereby exposing it to a greater risk of injury. The ACL in the narrow intercondylar fossa has flimsy morphology and biomechanics, and is thus easily damaged. Surgical evidence verifies that notchplasty can prevent the impingement between ACL and notch [12]. Several researchers suggested that notchplasty can be used as a routine procedure for ACL reconstruction, to prevent the graft from colliding with the intercondylar fossa and causing further injury [13]. However, the natural structure of the intercondylar fossa changes after enlargement, which may increase the risk of articular cartilage degeneration, biomechanical changes in patellofemoral joint, postoperative bleeding, and suboptimal rehabilitation. All these phenomena are not conducive to knee joint recovery activity and long-term clinical efficacy [14, 15]. Therefore, the preoperative evaluation of the femoral intercondylar fossa for ACL reconstruction can avoid excessive notchplasty.

Many measurement indices can be used to evaluate the morphology of femoral intercondylar notch, among which, the volume of the intercondylar fossa can well describe its complex 3D spatial configuration and has accurate results [16–18]. MRI is an important non-radiation method used to accurately measure the soft tissue and bone structure of the knee joint. Wratten and Zhang et al. calculated the 3D notch volume by manually tracking the boundary of notch on 2D MRI images; they concluded that the notch volumes were lower in ACL-injured individuals [7–9]. However, manual tracking of the boundary of each layer of the intercondylar notch on MR images is time-consuming and laborious and has limited efficiency, which hinder its clinical application. Therefore, a simple, fast, accurate and automatic image processing method should be developed to segment the intercondylar fossa and measure notch volume. The method can be used to predict and prevent the occurrence of ACL re-injury and guide the surgical design after the injury.

Deep learning has become a powerful tool for processing medical images due to its strong data self-learning ability and is widely used in various image segmentation tasks, including for brain tissue, bone, lung and blood vessels [19–24]. The segmentation function of deep learning can accurately locate the position of the target area and determine the shape and contour of the target by recognising the internal pixels or edges of region of interest (ROI) in medical images. The segmentation of cartilage, meniscus and ACL has been studied on the MRI of knee joint, and has satisfactory results in most cases [25–27]. At present, no deep learning-based technique has been developed for the segmentation of intercondylar notch.

This study aims to construct an automatic segmentation system of intercondylar fossa by using Res-UNet network. The system can automatically measure notch volume to compare its correlation with ACL injury.

## Methods

This retrospective observational study was approved by the Ethics Committee of our hospital. Participants’ records/information were anonymous and de-identified prior to analysis. Therefore, the requirement for written informed consent was waived.

### Subjects

**Fig. 1:**
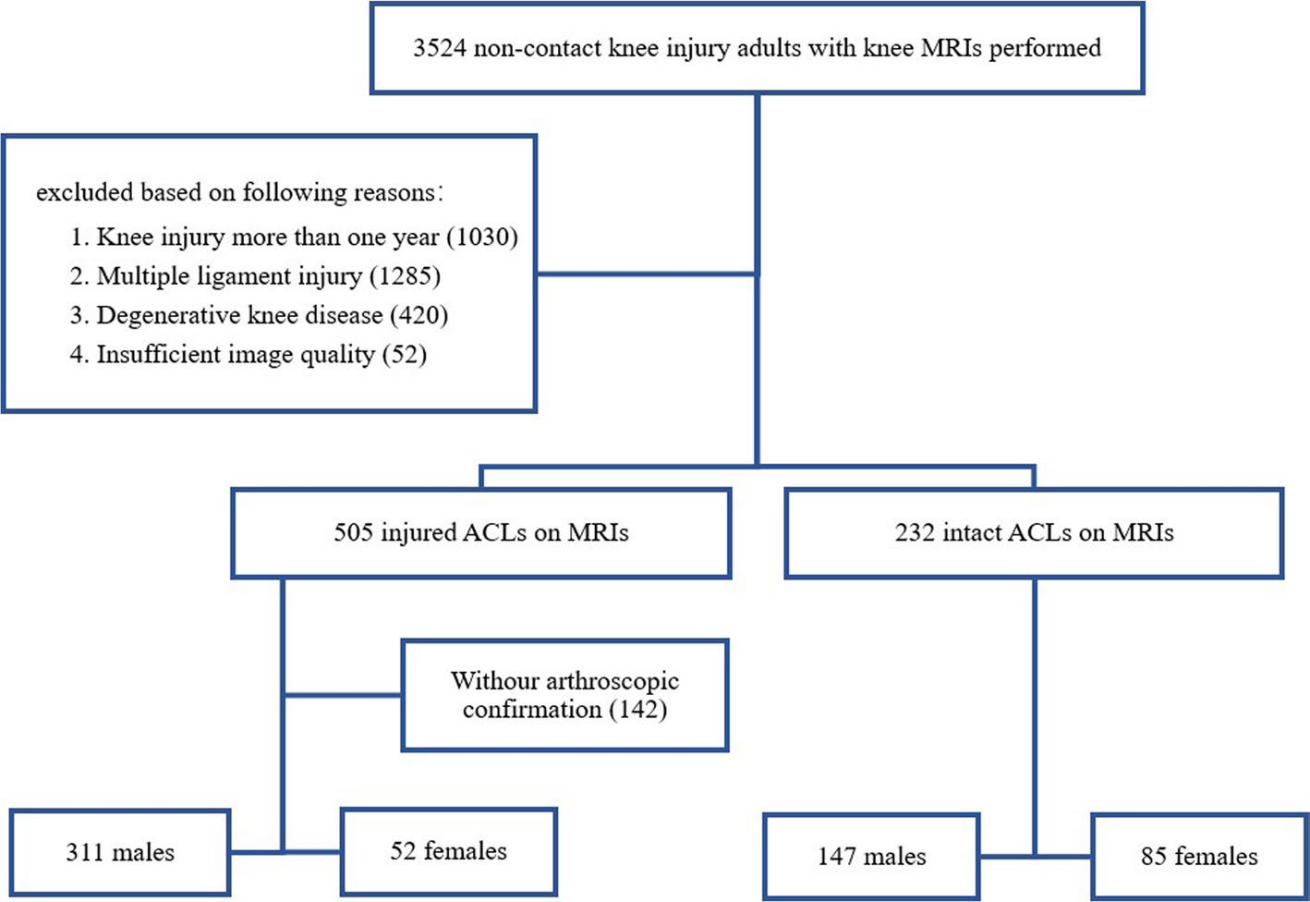
Flow diagram of patient recruitment

The MR images of patients who underwent MRI scan of knee joint from January 2016 to October 2020 were queried on the picture archiving and communication system (PACS) of the Third Affiliated of Southern Hospital by an experienced radiologist with 5 years of experience. The DICOM MR volumes of axial proton density- weighted spectral attenuated inversion recovery (PDW-SPAIR) (391 subjects) and axial T2-weighted spectral attenuated inversion recovery (T2W-SPAIR) (204 subjects) were collected from all subjects. A total of 3524 subjects age 18 to 60 years who had non-contact knee injury but had no knee surgery history were queried, and the flow diagram of patient recruitment is shown in Fig. 1. The non-contact knee injury included pivot shift injuries, axial load injuries or anterior translational injuries. Potential age-related changes in the femoral notch were ruled out. Juveniles were excluded because of their open epiphyseal plates, and the elderly were excluded because they had degenerative changes in joints. Patients with ACL injury for more than 1 year at the time of examination were excluded because knee joints with ACL defect degenerated faster than the normal ones; moreover, the proliferation of skeletal osteophytes may affect the calculation of the volume of the intercondylar fossa. Patients with multiple ligament injuries and degenerative knee diseases were also excluded. In addition, 52 notch volumes with poor signal-to-noise ratio or motion artifacts were excluded. Finally, 595 subjects were enrolled, including 363 patients with ACL injury and 232 patients without ACL injury. All ACL injury diagnoses were confirmed by arthroscopic pathology. All intact ACL diagnoses were confirmed by two senior radiologists after reading the MR images.

### MRI dataset and notch volume measurement

**Fig. 2:**
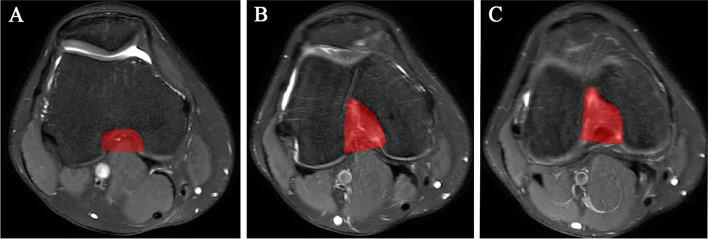
Axial slices of knee MRI showing the measurement of femoral intercondylar notch volume. **A** The most proximal level of the intercondylar notch. **B** One of the middle levels of the intercondylar notch. **C** The most distal level of the intercondylar notch

All subjects were scanned in the supine position with either a 1.5 T Achieva or 3.0 T Ingenia MR with an eight-channel knee coil (Philips Healthcare, Best, the Netherlands). The imaging parameters for the axial PDW-SPAIR sequence included the following: field-of-view (FOV) = 160 mm × 160 mm × 92 mm, echo time (TE) = 30 ms, relaxation time (TR) = 3000 ms, slice thickness = 4 mm and flip angle = 90°. The imaging parameters for the axial T2W-SPAIR sequence included the following: FOV = 160 mm × 160 mm × 105 mm, TE = 65 ms, TR = 2768 ms, slice thickness = 4 mm and flip angle = 90°. The MR images of different field intensities and sequences did not affect the analysis of the structure of the intercondylar fossa. The axial slices (thickness, 4.0 mm, slice gap, 0.4 mm) were selected to continuously display the edges of the femoral notch and calculate the volume. Similar to the method of Zhang et al. [9], we used ITK-SNAP software (v3.6; http://www.itksnap.org) to manually draw the boundary of each layer of the intercondylar notch according to the anatomical landmarks. The 2D notch area of each layer was calculated automatically by the software program. As shown in Fig. 2, the most proximal boundary of the intercondylar notch was defined as the level where the femoral condyles and their cartilages were clearly visible (Fig. 2A). The most distal boundary was defined as the last continuous layer of the condyle where the femoral condyles were anteriorly continuous (Fig. 2C). Figure 2B was one of the middle levels of the intercondylar notch. Notch volume was calculated by summing all the 2D areas and multiplying its slice thickness plus slice gap.

All measurements were conducted by an experienced radiologist with 5 years of experience. Volume measurements were repeated twice by the same radiologist at a 3-month interval to assess intra-observer reliability. Measurements were made by another radiologist with 6 years of experience to assess inter-observer reliability.

### MRI dataset preprocessing

All MR images of 595 patients were trained together as experimental subjects. Our segmentation model requires two inputs: the original MR images and the ROI of the outlined intercondylar fossa. Preprocessing steps was performed on the original MR images. The specific steps were as follows:

- To avoid the overfitting problem, we randomly rotated ± 10°, shifted ± 20 voxels in the x and y coordinates and horizontally flipped each MR images to increase the training set size.
- Image preprocessing included resizing all the images into 512 × 512 to ensure uniform input dimensions, as well as histogram matching, and rescaling to [0,1].
- The intercondylar fossa was displayed in several layers of the MR images. To reduce the influence of irrelevant image layers on the segmentation accuracy, we only extracted the number of layers with ROI for training.

### Segmentation model

**Fig. 3:**
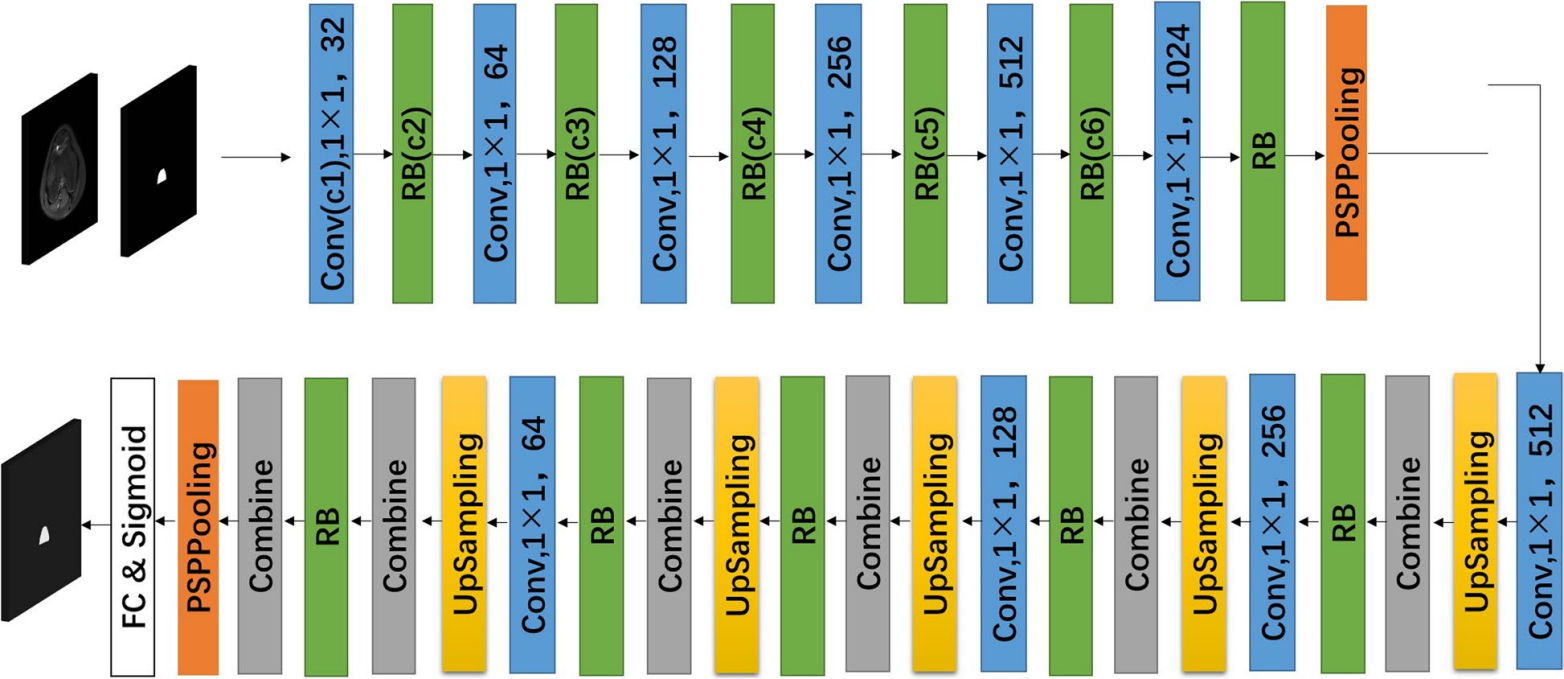
Illustration of the segmentation model. The U-Net network architecture is structured into an encoder and a decoder. The encoder follows the classic architecture of the convolutional neural network, with each convolutional blocks followed by a rectified linear unit (ReLU) and a maximum polling operation to encode image features at different levels of the network. The decoder up-samples the feature map with subsequent up-convolutions and concatenations with the corresponding encoder blocks. The network has two inputs: the preprocessed original MR images and the outlined ROI of the intercondylar fossa. The segmentation architecture consists of 10 convolutional layers, 11 residual blocks (RBs), two pyramid pooling modules (PSPPooling), five upsampling layers, six combine blocks and a fully connected (FC) layer, and a sigmoid layer. Conv = convolution, RB = residual block, PSPpooling = pyramid scene parsing pooling, FC = fully connected

In this study, we employed semantic segmentation using Res-UNet [28], which was constructed based on the architecture of U-Net [29]. Residual blocks were introduced in the model [30], and its organization is shown in Fig. 3. The architecture of the network helps localise and extract image features and assemble a precise output based on encoder information. The addition of residual modules solves the problem of difficulty in training caused by network depth. Our experiments confirmed that the network outperforms other popular convolutional neural network models.

#### Residual block

**Fig. 4:**
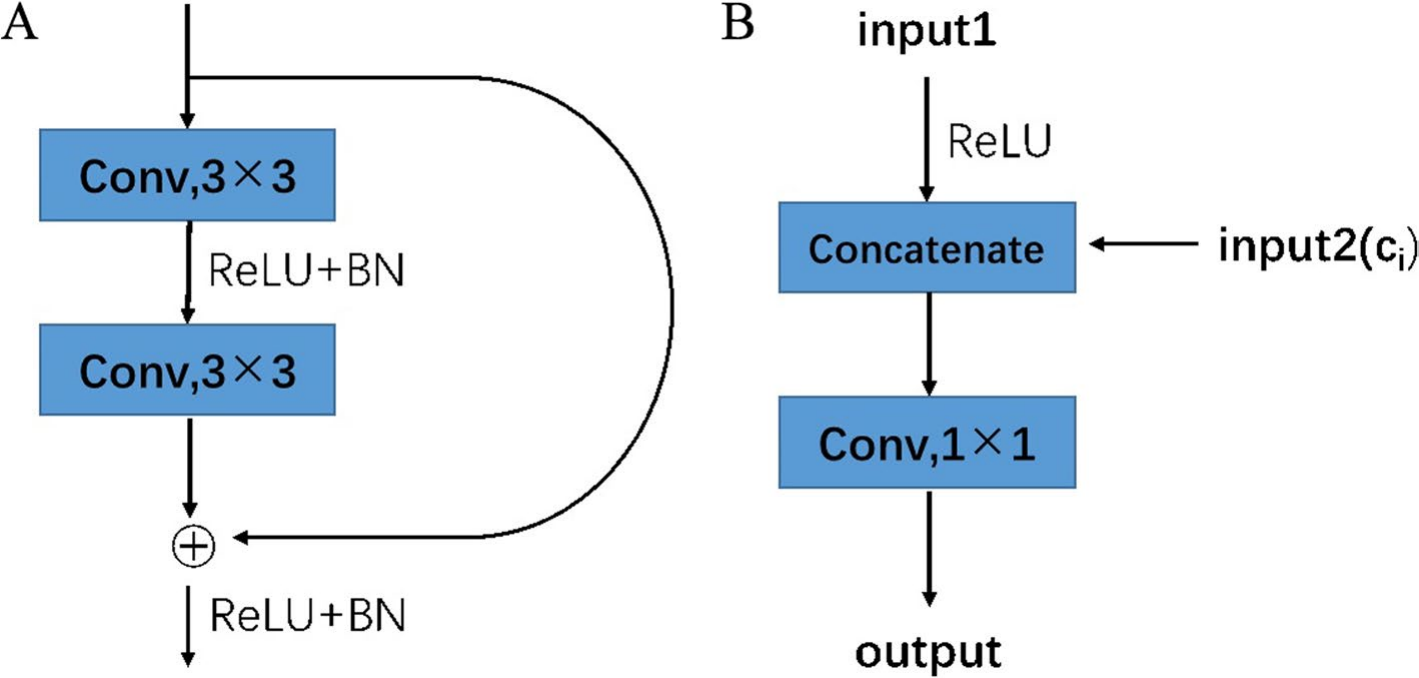
**A** Structure of the residual block. Each residual block (RB) consists of two batch normalization layers, two rectified linear unit(ReLU)layers and two 3 × 3 convolutional layers. **B** The structure of the combine block. Each combine block consists of two inputs, a rectified linear unit(ReLU)layer and a convolutional layer

As shown in Fig. 4 (A), each residual block (RB) consists of two batch normalizations layers, two rectified linear unit (ReLU) layers and two 3 × 3 convolutional layers.

#### Combine block

The feature map obtained by each convolutional layer of the network is concatenated to the corresponding up-sampling layer, so the feature map of each layer can be effectively used in subsequent calculations. It is a common skip-connection process. Compared with other network structures, such as FCN [31], combine block avoids direct supervision and loss calculation in the high-level feature map but combines the features in the low-level feature map. Therefore, the final feature map can contain high-level features and many low-level features. This property allows the integration of features under different scales and improves the accuracy of the model results. The organization of the model is shown in Fig. 4 (B).

#### Training loss

A dice loss function is specifically designed to mitigate dataset class imbalance and is frequently used for medical imaging segmentation. The dice similarity coefficient (DSC) is measured as an overlap of the output mask with ground truth to assess each segmentation task. DSC measures the overlap between set X (the ground truth) and Y (the predicted mask). For binary class segmentation, the DSC is expressed as follows:

$$\mathrm{D}\text{SC = }\frac{2*\left|\mathrm{X}\cap \mathrm{Y}\right|}{\left|\mathrm{X}\right|+\left|\mathrm{Y}\right|}$$

$$\mathrm{DiceLoss}\text{ = 1}-\frac{2*\left|\mathrm{X}\cap \mathrm{Y}\right|}{\left|\mathrm{X}\right|+\left|\mathrm{Y}\right|}$$

### Implementation

The segmentation network was coded in Python 3.6 by using open-source Tensorflow packages. Training was performed on a GPU-optimized workstation with a single NVIDIA GeForce RTX 2080 Ti. The training time for the proposed segmentation model was 30 h. The parameters were set as follows: a Stochastic Gradient Descent (SGD) + momentum optimizer with a learning rate of 1e-4. We trained a total of 50 epochs and used dice loss for our training loss.

We randomly divided the data set into 534 and 61 volumes for training and test sets, respectively. In the training phase, a fivefold cross-validation paradigm was used, in which 80% of the data were randomly allocated to the training queue, and the remaining 20% were used for validation. The process was repeated five times until each exam in the entire training data set was used for validation once. Finally, the trained network model was applied to the test set to obtain the experimental results. In addition, the axial MR images of 30 patients from the Longgang Central Hospital of Shenzhen were selected as an external test dataset for testing to verify the reliability of the results. The inclusion and exclusion criteria of patient images are the same as above. The cumulative verification set statistics of the entire training data set are reported below.

After the segmentation results were obtained, the area (*s*) of each layer was calculated according to the number of pixels. The sum of the area multiplied by the slice thickness plus slice gap (d) of the MR images is the volume (V) of the intercondylar fossa. The volume is expressed as follows:

$$V=\sum_{1}^{n}sd$$

n is the number of layers of the intercondylar fossa in the MR images.

### Model performances evaluation and statistical analysis

All values are shown as mean ± SD. Intraclass correlation coefficient (ICC) was determined to analyze the intra- and inter-observer reliability for the volume measurements. ICC values < 0.4, 0.4–0.75, and > 0.75 represent poor, moderate, and good repeatability, respectively. The segmentation performance of the models was evaluated using DSC. The numerical range of DSC is 0–1, and higher value indicates better segmentation effect. Pearson correlation and Bland–Altman analysis were used to evaluate the ability of the model for automatic segmentation and manual segmentation. Unpaired, two-tailed t-test was performed to determine differences in automatically measured notch volume between the ACL-injured group and the intact group as well as between males and females. All the statistical analyses were performed by Statistical Product and Service Solutions for Windows (SPSS, version 22.0, USA).

## Results

### Automatic segmentation performance

The intra- and inter-observer reliability for the volume measurements showed good repeatability. The ICC for intra-observer reliability is 0.981 (0.967, 0.989), and that for inter-observer reliability is 0.972 (0.947, 0.985).

**Fig. 5:**
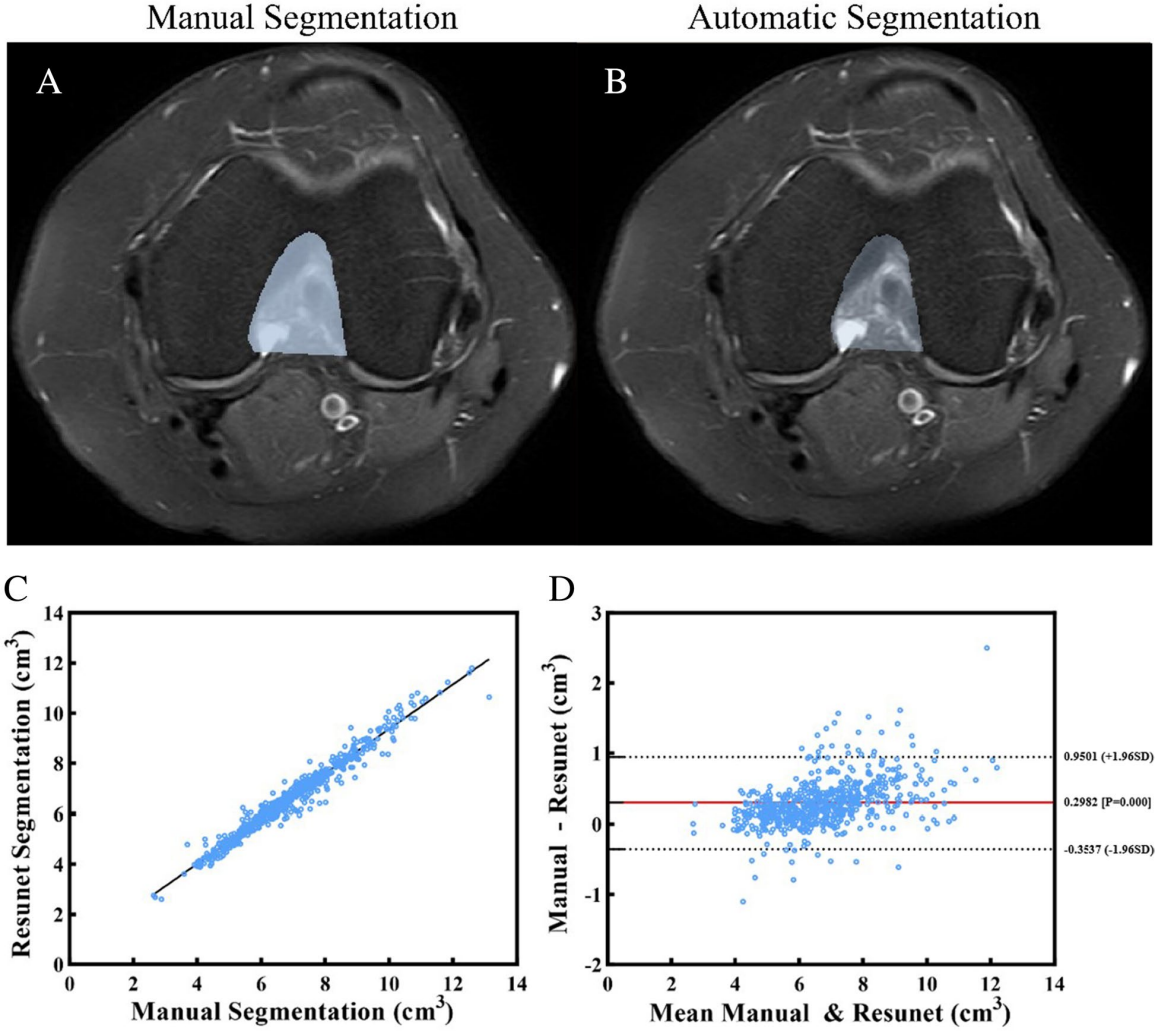
MR images showing comparison between, **A** manual segmentation and **B** automatic segmentation predicted using the Res-UNet convolutional neural network. **C** Scatterplots and **D** Bland–Altman plots showing the comparison of volume calculations from the manual and automatic segmentation methods

Figure 5 shows the comparison of the sections predicted from the manual segmentations of the femoral intercondylar notch volume of the test data sets (Fig. 5A) and from the automatic segmentation (Fig. 5B). As shown in the scatterplots (Fig. 5C) and Bland–Altman plots (Fig. 5D), the volume of the data-sets between manual and automatic segmentations showed strong linear relationships and correlation across intercondylar notch, with *R*^2^ values of 0.9647.

We compared our method with U-Net with other methods. Table 1 shows that the mean DSCs calculated for predicting the femoral intercondylar notch volume in the data-set by using Res-UNet is 0.916 ± 0.04, while the relative error is 0.047 ± 0.036. Similar results were obtained in the external test dataset (Table 2). Hence, the proposed method performed better than other methods such as U-Net, Seg-Net [32], Dense-UNet [33], and Mobile-UNet [34].

**Table 1:** Average Results for 5-Fold Cross-Validation with Different Networks

Network	Dice similarity coefficient	Automatic Segmentation Volume (cm^3^)	Manual Segmentation Volume (cm^3^)	Relative Error
U-Net	0.914 ± 0.04	6.483 ± 1.500	6.874 ± 1.644	0.061 ± 0.037
Seg-Net	0.906 ± 0.10	6.384 ± 1.484	6.874 ± 1.644	0.072 ± 0.036
Res-UNet	0.916 ± 0.04	6.576 ± 1.492	6.874 ± 1.644	0.047 ± 0.036
Dense-UNet	0.901 ± 0.08	6.347 ± 1.474	6.874 ± 1.644	0.077 ± 0.035
Mobile-UNet	0.906 ± 0.07	6.312 ± 1.452	6.874 ± 1.644	0.085 ± 0.051

**Table 2:** Results for the external test dataset with Different Networks

Network	Dice similarity coefficient	Automatic Segmentation Volume (cm^3^)	Manual Segmentation Volume (cm^3^)	Relative Error
U-Net	0.914 ± 0.04	7.206 ± 1.497	7.421 ± 1.476	0.064 ± 0.018
Seg-Net	0.906 ± 0.10	7.184 ± 1.498	7.421 ± 1.476	0.071 ± 0.016
Res-UNet	0.916 ± 0.04	7.251 ± 1.492	7.421 ± 1.476	0.054 ± 0.019
Dense-UNet	0.901 ± 0.08	7.169 ± 1.490	7.421 ± 1.476	0.075 ± 0.016
Mobile-UNet	0.906 ± 0.07	7.178 ± 1.516	7.421 ± 1.476	0.078 ± 0.013

The relative error is expressed as the following: $E=\frac{R-P}{R}$ R is the real volume and P is the predicted volume

The processing of the data of each patient's intercondylar fossa to generate a single subject volume took 10 min for manual segmentation but only 3–5 s for automatic segmentation, which is very time-saving.

### Measurement of the femoral intercondylar notch

The mean value of the automatically measured intercondylar notch volumes in the ACL-injured and ACL intact groups are shown in Table 3. The notch volume was significantly lower in the ACL-injured cohort than in the intact cohort (6.12 ± 1.34 cm^3^ vs. 6.95 ± 1.75 cm^3^, *p* < 0.001). Females had predisposition for smaller femoral notches than males (5.41 ± 1.30 cm^3^ vs. 6.76 ± 1.51 cm^3^, *p* < 0.001).

**Table 3:** Mean intercondylar notch volume of ACL-injured, ACL intact, male and female participants

	Age (years)	Notch volume (cm^3^)	*P* value
Injured ACL	30.76 ± 8.08	6.12 ± 1.34	< 0.001
Intact ACL	33.80 ± 11.74	6.95 ± 1.75	
Males	30.75 ± 8.44	6.76 ± 1.51	< 0.001
Females	35.95 ± 12.54	5.41 ± 1.30	

The differences in notch volume between males and females are shown in Table 4. The notch volumes of males and females with injured ACL were 6.33 ± 1.25 and 4.89 ± 1.23 cm^3^, respectively, whereas those with intact ACL were 7.66 ± 1.61 and 5.73 ± 1.25 cm^3^, respectively. Males and females who had ACL injuries had smaller notch than those with intact ACL (*p* < 0.001 and *p* < 0.005). Moreover, men had larger notches than women, regardless of the ACL injuries (*p* < 0.001).

**Table 4:** Mean intercondylar notch volume of male and female ACL-injured and ACL-intact groups

Notch volume (cm^3^)	Gender		*P* value
	Males	Females	
Injured ACL	6.33 ± 1.25	4.89 ± 1.23	< 0.001
Intact ACL	7.66 ± 1.61	5.73 ± 1.25	< 0.001
*P* value	< 0.001	< 0.005	

## Discussion

Previous studies observed that the notch volumes in the ACL injury group were significantly lower than those in the normal ACL group [7–10]. This finding indicates that the stenotic intercondylar fossa is a risk factor for ACL injury, and appropriate notchplasty can avoid this risk. It is useful to evaluate the shape of the intercondylar fossa before ACL reconstruction, though the characteristics of narrow notches are still controversial. To evaluate the correlation between notch volume and ACL injury, we introduced an automatic intercondylar fossa segmentation network based on deep learning. The network can quickly and accurately segment the intercondylar fossa automatically and quantify the notch volume. Although the training time of the network is relatively long, the segmentation efficiency is very high once the network training is completed. This significant improvement in efficiency will lead to rapid extraction of information and efficient application in research.

Previous studies compared notch volume between adults with ACL injury and intact ACL. However, the results are conflicting, and the notch volume is not the same. Van Eck et al. is the first to use 3D volume measurement technology to determine the relationship between ACL injury and intercondylar fossa size [35]. Although this study used the same methods as in the present work, the results are contradicting; they found that the notch volume in subjects with injured ACL was lower than that in subjects with normal ACL, and the difference was not statistically significant (*P* = 0.052). The reason for the difference in the finding is unclear but may be related to the small sample size and the imbalance of gender ratio in the study cohort. The notch volumes measured by Wratten and Zhang were smaller than those in the present work [7–9]; in particular, in the study of Wratten, the notch volume were lower than those in the study on young people with immature bone [36]. The discrepancy in the results may be related to the diversity of femoral anatomy due to ethnic differences. The frequency of ACL injury in women is higher than that in men while performing the same exercise. The possible reasons include external and internal factors, such as motor skills, physiological hormones, and smaller knee in women [2, 37], consistent with the fact that men have larger whole skeleton. However, the majority of patients with ACL injury in the present study were males. Males were involved in more intense exercise, such as football and basketball, than females, leading to an increased risk of injury. In addition, the hospital where the images were obtained is a designated hospital for football players; as such, a certain bias in admission rate may exist, resulting in more male patients. Since we didn’t analyze the relationship between the notch volume and the demographic factors, such as height and weight, the gender differences caused by all these demographic factors could not be ruled out.

Deep learning-based segmentation methods do not need prior knowledge of structure shape and can provide accurate segmentation in the presence of image artifacts. They are suitable for the segmentation of structures without normal shape reference. The deep learning algorithms commonly used in medical image segmentation include U-Net [29], Res-UNet [28], Seg-Net [32], Dense-UNet [33], and Mobile-UNet [34]. In the present study, we used Res-UNet to construct an intercondylar fossa segmentation model based on U-Net architecture and introduced residual blocks in the model. The introduction of the residual structure allows the construction of a segmentation network with deeper network layers and maintains high training efficiency, thereby improving the segmentation accuracy [28].

The accuracy of automatic segmentation of the intercondylar fossa is close to that of manual labelling, and the DSC value is more than 0.90, indicating the good performance of the system. The results of automatic segmentation of the intercondylar fossa can help clinicians predict the probability of ACL injury and re-injury after surgery [4–6]. When the notch volume is small, preventive training during strenuous exercise is recommended. Notchplasty can also be considered during ACL reconstruction.

This study has some key limitations. Firstly, this retrospective study has inherent biases. Further prospective multi-institutional research is warranted to determine the applicability of the proposed detection system. Secondly, not all subjects in the control group are normal subjects, and may have meniscus injury, synovitis or tendon injury, which may lead to bias. To avoid evaluating notch volume changes caused by other diseases, we excluded patients with bone defect, bone erosion and bone hyperplasia, such as osteochondritis exfoliation, villonodular synovitis, and obvious degenerative changes in joint. Thirdly, our research subjects are Chinese people aged 16 to 60. We excluded teenagers, elderly, and patients who underwent knee surgery. Therefore, our conclusion does not apply to all patients. Fourthly, we could not make corrections for anthropometric factors due to missing information about the height, weight, and other demographic factors of the participants. Gender differences caused by these demographic factors could not be ruled out and need to be further verified by future research.

## Conclusions

In this study, we firstly published a deep learning model that automatically measured intercondylar notch with high precision. The model such this saves huge amount of time in measuring notch volume, which is helpful to predict and prevent ACL injury and to evaluate the necessity of notchplasty before ACL reconstruction to prevent ACL re-injury.

## Data Availability

The datasets used and/or analysed during the current study are de-identified and available from the corresponding author on reasonable request.

## Abbreviations

ACL: Anterior cruciate ligament
ROI: Region of interest
PACS: Picture Archiving and Communication System
PDW-SPAIR: Proton Density Weighted Spectral Attenuated Inversion Recovery
T2W-SPAIR: T2-Weighted Spectral Attenuated Inversion Recovery
ReLU: Rectified Linear Unit
RBs: Residual Blocks
PSPPooling: Pyramid Pooling Modules
FC: Fully Connected
DSC: Dice Similarity Coefficient
SGD: Stochastic Gradient Descent
ICC: Intraclass Correlation Coefficient
